# Epilepsy in Pregnancy—Management Principles and Focus on Valproate

**DOI:** 10.3390/ijms23031369

**Published:** 2022-01-25

**Authors:** Barbara Błaszczyk, Barbara Miziak, Ryszard Pluta, Stanisław J. Czuczwar

**Affiliations:** 1 Faculty of Medical Sciences, High School of Economics, Law and Medical Sciences, 25-734 Kielce, Poland; barbarablaszczyk@op.pl; 2 Department of Pathophysiology, Medical University of Lublin, 20-090 Lublin, Poland; barbara.miziak@umlub.pl; 3 Laboratory of Ischemic and Neurodegenerative Brain Research, Mossakowski Medical Research Institute, Polish Academy of Sciences, 02-106 Warsaw, Poland; pluta@imdik.pan.pl

**Keywords:** antiepileptic drugs, pregnancy, congenital malformations, neurodevelopmental disorders, histone deacetylase, valproate

## Abstract

An estimated 60 million people worldwide suffer from epilepsy, half of whom are women. About one-third of women with epilepsy are of childbearing age. The childbirth rate in women with epilepsy is about 20–40% lower compared to that of the general population, which may be partly due to a lower number of these women being in relationships. Lower fertility in women with epilepsy may be linked to the disease itself, but it is mainly a result of the treatment provided. Valproate, as an antiepileptic drug inhibiting histone deacetylases, may affect the expression of genes associated with cell cycle control and cellular differentiation. Evidently, this drug is associated with the risk of malformations although other antiepileptic drugs (AEDs) may also trigger birth defects, however, to a lower degree. Valproate (and to a certain degree other AEDs) may induce autism spectrum disorders and attention deficit hyperactivity disorder. The main mechanism responsible for all negative effects of prenatal exposure to valproate seems inhibition of histone deacetylases. Animal studies show a reduction in the expression of genes involved in social behavior and an increase in hippocampal cytokines. Valproate-induced oxidative stress may also contribute to neural tube defects. Interestingly, paternal exposure to this AED in mice may trigger neurodevelopmental disorders as well although a population-based cohort study does not confirm this effect. To lower the risk of congenital malformations and neurodevelopmental disorders, a single AED at the optimal dose and supplementation with folic acid is recommended. VPA should be avoided in women of childbearing age and especially during pregnancy.

## 1. Introduction

Prospective studies in the past 10 years have revealed an increased risk of infertility in women, especially those receiving polytherapy for epilepsy [1]. Infertility affects 7.1% of women not using antiepileptic drugs (AEDs), 31.8% treated with monotherapy, 40.7% treated with two drugs, and 60.3% treated with three or more AEDs [1]. Polycystic ovary syndrome (PCOS), especially observed after the use of valproic acid (VPA) in women under 26 years of age may account for fertility problems [2]. One in six women with epilepsy live in India, accounting for 2.73 million patients in the country, 52% of whom are of reproductive age (15–49 years) [1]. In the US, approximately 1.5 million women with epilepsy are of childbearing age and give birth to approximately 25,000 children per year, that is, 3–5 children per 1000 born [3]. The results obtained by Pennel et al. [4] may indicate that primary causes of reduced fertility seem social rather than biological. They evaluated 197 women—89 with epilepsy and 108 as a control group. Among women with epilepsy, 60.7% achieved pregnancy and among the control group—60.2% within 21 months. 

Statistically, 0.3–0.5% of women giving birth suffer from epilepsy. In Poland, this affects approximately 1800 women per year. The incidence of new cases of epilepsy diagnosed in women of childbearing age is 20–30/100,000/year. Both epilepsy and the administered pharmacotherapy present an increased risk to a pregnant woman and the fetus. This higher risk for a woman with epilepsy is caused by the physiological, metabolic, and hormonal changes that occur during pregnancy, which alter the pharmacokinetics of the drugs and may result in seizures. This presents both a risk of obstetric complications and a threat of abnormal fetal development [5].

The risk to a fetus carried by an epileptic woman stems from the teratogenic effect of AEDs, leading to congenital malformations and dysmorphia, which may occur mainly in the first trimester of pregnancy, as well as neurodevelopmental deficits and subsequent behavioral problems resulting from hypoxia during seizures [6]. Planning pregnancy is the most optimal and safest method for both the woman with epilepsy and her future child. This allows for optimization of pharmacotherapy in order to reduce the risk of fetal anomalies on one hand and, on the other, to best control seizures during pregnancy, which reduces the risk of subsequent developmental disorders in the child. However, the US studies indicate that 50% of pregnancies are not planned, and 40% of patients diagnosed with epilepsy do not consult a physician before becoming pregnant [6,7]. Although the risk of pregnancy complications is higher in women with epilepsy than in the non-epileptic ones, more than 90% of pregnancies have a normal course, and these women deliver healthy babies [5]. 

## 2. Preconception Care in Women with Epilepsy

The management principles during pregnancy in epileptic women are addressed in numerous publications and recommendations. They are included inter alia in the guidelines of the Polish Society of Epileptology and Polish Gynecological Society, by Jedrzejczak et al. [5]. Childbearing-aged women with epilepsy should be informed that epilepsy is not a contraindication to giving birth. Over 90% of women with epilepsy deliver healthy children. Pregnancy in women with epilepsy, as already mentioned, should be planned and the woman should prepare for the pregnancy in a conscious manner [8]. The risk of congenital malformations in children of mothers with epilepsy depends on the type of administered drug, the number of drugs, and the dosage—it is higher than in the general population and reaches 3% in those using carbamazepine (CBZ) or lamotrigine (LTG); 7% for valproate (VPA); 15% in those receiving two or more AEDs [9]. Since VPA is a frequently used AED, it is important to assess the benefits and risks of its application on a case-by-case basis [9]. Epilepsy is not a contraindication to natural childbirth, epidural anesthesia during labor, or breastfeeding. Women with epilepsy who decide not to have children may receive effective contraception [5].

The key element in the care of women with epilepsy of childbearing age is pregnancy planning. While the number of planned pregnancies is rising as a result of increased awareness and knowledge of epilepsy, 40% are still unplanned. The risk of major fetal malformations is higher in women with epilepsy taking AEDs [9,10]. The concerns of a woman with epilepsy about the adverse effects of AEDs on her child may prompt her to arbitrarily discontinue or reduce doses of AEDs, increasing the risk of seizures and even resulting in SUDEP syndrome (sudden unexpected death in epileptic patients) [11,12,13]. It is therefore important to promote early care of women of childbearing age (15–44 years) who are treated for epilepsy by specialized neurologic and gynecological clinics [5,14].

Providing care for women with epilepsy in the preconception period is intended to reduce the risk of fetal abnormalities and subsequent disorders of child development by optimizing pharmacological treatment and implementing folate supplementation. This may also provide desirable seizure control during pregnancy. It is important to explain the issue to the patient and her relatives and to emphasize that a potential teratogenic factor, such as the use of AEDs, is already present in the first days after conception. To reduce the risk of birth defects, it is recommended to administer the lowest effective dose of the most appropriate AED until at least the end of the first trimester of pregnancy [5,9]. Discontinuation of medication may be considered in women after a three-year seizure-free period [5]. Pregnant women who have had seizures a year before conception require increased monitoring of their epilepsy treatment. Most women (67%) do not experience seizures during pregnancy. Between 74% and 92% of women who have been seizure-free for at least 9 months to 1 year before pregnancy will remain free of seizures during pregnancy [5,15,16]. The data from EURAP (European Registry of Antiepileptic Drugs and Pregnancy) have shown that pregnant women with idiopathic generalized epilepsy are more likely to be free of seizures (74%) than those with focal epilepsy (60%) [11]. 

Women with epilepsy should be informed that implementing a few safety guidelines can significantly reduce the risk of seizures and minimize anxiety about the effects of AEDs on the child [17,18,19]. Preconception period recommendations follow:

- Every woman with epilepsy during preconception should be provided with comprehensive information on the course of epilepsy during pregnancy.
- If treatment with AEDs is necessary, monotherapy should be used if possible.
- If a change of an AED is necessary, it should be done before pregnancy.
- A woman planning pregnancy should take folic acid (5 mg/day) before and during the early stages of pregnancy.
- Women using AEDs that induce liver enzymes may receive vitamin K during the last period of pregnancy.
- All pregnancies of women with epilepsy should be reported to an appropriate epilepsy and pregnancy registry [12].

## 3. Epileptic Seizures in Pregnancy

The first-ever epileptic seizure can occur at any time during pregnancy. These may be symptomatic seizures occurring as a result of metabolic disorders, low blood pressure, eclampsia, and other conditions coinciding with pregnancy such as central nervous system (CNS) infections or strokes. The first epileptic seizure can occur at any time during pregnancy as a consequence of vascular malformations [13,20].

## 4. Frequency of Epileptic Seizures during Pregnancy 

Most women with epilepsy do not have seizures during pregnancy, whereas a third of them may experience an increase in the number of epileptic seizures occurring during this period [21]. The data from the Epilepsy Pregnancy Registry report that seizures are not observed in 50–67% of women [22,23,24]. The EURAP study covering 42 countries and involving 3784 pregnant women with epilepsy found that 66.6% were seizure-free, with 58.2% treated with LTG, 75% with VPA, 67.35% with CBZ, and 73.4% with phenobarbital (PB). Generalized tonic-clonic seizures were more frequently observed with LTG treatment (21.1%) than with VPA (11.5%), CBZ (12.6%), or PB (14.0%) [13].

With the planned pregnancy, seizures are less frequent, treatment is more often managed with a single drug, and VPA is used less frequently [13]. The occurrence of at least two epileptic seizures for the first time in life, or the recurrence of epileptic seizures after a long break, is an indication to initiate AED therapy during pregnancy. If epileptic seizures were rare before pregnancy, the dose of an AED may be reduced. However, if the frequency of seizures has risen during pregnancy, it is necessary to increase the dose of the drug [9]. An increase in the frequency of epileptic seizures may result from the fact that during pregnancy the plasma volume grows, the binding of drugs to plasma proteins is altered and the clearance of AEDs is also changed, which may reduce their bioavailability. It is therefore recommended that concentration levels of an AED in serum be determined during pregnancy [25].

During pregnancy, the serum concentration of AEDs tends to drop in different degrees. It is at 50–60% of the pre-pregnancy level for LTG, levetiracetam (LEV)—40–60%, oxcarbazepine (OXC)—30–40%, topiramate (TPM)—30–40%, and zonisamide (ZSM)—20–40% [26,27]. Most women using LTG or LEV during pregnancy may require an increased drug dose [28]. In addition to pharmacokinetic factors, physiological factors (e.g., sleep deprivation or disruption, nausea, vomiting, nycturia), metabolic factors (increased sodium and water retention), hormonal factors (changes in estrogen and progesterone levels, increased gonadotropin levels in the first trimester of pregnancy), and psychological factors (stress, anxiety, fear of adverse effects of medication on the child, non-compliance with recommendations) also contribute to the increased frequency of epileptic seizures in pregnant women [29,30]. The number of epileptic seizures occurring before pregnancy may suggest difficulties in seizure control during pregnancy. The occurrence of seizures one month before pregnancy increases the risk of seizures during pregnancy 15-fold [13]. One of the consequences of epileptic seizures during pregnancy may be premature placental detachment or fetal hypoxia [31].

## 5. Teratogenicity of AEDs 

If women with epilepsy must receive AEDs during pregnancy, it is safer for the mother and her child to use the AED with low teratogenicity and at the lowest effective doses possible. The risk of congenital malformations in children of women with epilepsy not using AEDs is found in the range of 1.1–3.3% and is similar to the risk of malformations in the general population (2.1–2.9%) [32]. The risk of congenital defects in children of women with epilepsy and using AEDs is 4–9% and increases with VPA and polytherapy [33,34,35,36,37].

It has been reported that monotherapy with newer AEDs such as LEV [35,38], LTG [35], OXC, and ZSM [39,40] is relatively safe for pregnant women. LEV and LTG are considered safe for pregnancy and their use has not been associated with an increased risk of birth defects, compared with the control group [35,41]. Currently, there are not yet sufficient data (small number of pregnant women surveyed) to assess the risk of birth defects for other AEDs used in monotherapy, such as gabapentin (GBP), lacosamide (LCM), OXC, pregabalin (PGB), or TPM [9]. However, the current data on the use of new-generation AEDs, while still limited, indicate a more favorable safety profile and lower teratogenic risk than older drugs [42]. The incidence of defects in children of mothers treated with AEDs depends not only on the type of AED but also on its dose. The risk of a defect increases with higher doses of a drug [43]. The EURAP registry assessed the incidence of congenital malformations manifesting up to 1 month of age and then those diagnosed later up to 1 year of age [11,44]. Teratogenic effects of the most commonly used AEDs are shown in Table 1.

## 6. Teratogenicity Risk of AEDs

Mechanisms of fetal congenital malformations include: conversion of AEDs to toxic, unstable metabolites and folic acid deficiency with subsequent increase in homocysteine levels (see below) [25].

## 7. AED Polytherapy and the Risk of Major Birth Defects in Children of Mothers with Epilepsy

Using several AEDs increases the risk of more severe birth defects in the fetus. Depending on the combination of AEDs, the possible teratogenic effects may vary. Observations show a higher risk of birth defects with polytherapy that combines AEDs with VPA. The combinations with LEV or LTG appear to be safer (see below) [35].

## 8. Types of Congenital Malformations in Children of Women with Epilepsy Using AEDs

The most commonly reported congenital malformations in children of women using AEDs are: cleft palate, neural tube defects, skeletal abnormalities, and congenital heart and urinary tract defects [45]. Some defects are more commonly found with the use of particular AEDs. Thus, PB seems more likely to induce the development of heart defects than phenytoin (PHT) or CBZ [25,46]. The use of VPA is found to be associated with an increased incidence of spina bifida, cleft palate, cardiac abnormalities, and hypospadias, among other things [25,47,48]. The occurrence of cleft palate is more frequently observed with the use of PB, VPA, or TPM [21,25].

## 9. Folic Acid Supplementation

Clinical studies have shown that folic acid supplementation can prevent malformations in children of women taking AEDs, hence the recommendation of a dose of 5 mg/day of folic acid for women considering pregnancy and during the first months of pregnancy [49,50]. High doses of folic acid (above 5 mg/day) are not recommended as they may lower the seizure threshold.

## 10. Effects of AEDs Use in Pregnant Women on Their Children’s Cognitive Functions

The use of AEDs by women with epilepsy during pregnancy may affect their children’s intellectual development in the future. It has been found that the use of VPA by women during pregnancy may affect their children’s cognitive functions and lead to IQ decline [9,51]. The use of PHT and PB or polytherapy also has a negative effect on the intellectual development of children of epileptic mothers, while no such negative effect was observed with the use of CBZ [5]. Multicenter analyses have shown that children of mothers using VPA during pregnancy, surveyed at the age of six, had lower IQs than children of mothers using CBZ, LTG, or even PHT [9]. However, children of mothers who received folic acid supplementation during pregnancy had higher IQs than those whose mothers did not receive it [9].

It should be particularly underlined that the children of mothers using VPA during pregnancy were also found to display a higher incidence of autism spectrum disorders (ASD) and attention-deficit hyperactivity disorder (ADHD) symptoms (see below) [2,52,53,54].

## 11. Monitoring Serum Levels of AEDs

Serum concentrations of most AEDs may fluctuate in pregnancy due to changes in pharmacokinetics during absorption, metabolism, or excretion. It has been found that concentrations of LTG, LEV, or OXC in pregnant women can decrease by up to 30–50% [5,9]. Lower drug concentrations may lead to intensified seizures. It is therefore recommended to monitor the serum levels of these drugs before pregnancy and at least once during each trimester of pregnancy and additionally in special situations such as lack of seizure control or the occurrence of adverse symptoms [25]. Considering LTG, this AED penetrated into amniotic fluid with a ratio of 0.68, umbilical cord (penetration ratio—0.92), and breast milk (0.77) [28]. Again, these data may recommend therapeutic drug monitoring [28]. A double-blind randomized trial was carried out in order to compare two strategies in pregnancy: therapeutic drug monitoring or clinical features monitoring [55]. In the first strategy, clinicians could obtain monthly serum AED concentrations, also having access to clinical data. In the second strategy, adjustment of AED dosage (CBZ, PHT, LTG, LEV) was only based on clinical findings. The results clearly indicate that after randomization, the time to first seizure or timing of all seizures did not differ significantly in both groups. Also, maternal and neonatal outcomes were comparable, the only exception being higher cord blood concentrations of LTG and LEV. However, increased exposure to these AEDs did not result in any maternal or fetal complications. In the light of these results, therapeutic AED monitoring in pregnant women with epilepsy may not be indispensable [55].

## 12. Neuropsychiatric Disorders of Women with Epilepsy in Pregnancy

It is important to note that symptoms of depression, depressed mood, poor concentration, fatigue, irritability, or anger are more common in pregnant women using AEDs [5].

## 13. Antenatal Care during Pregnancy

When providing care for pregnant women with epilepsy, physicians should be aware of a possible minor risk increase in complications during pregnancy, particularly in women taking AEDs [5,16,56]. A 2015 review of 38 studies involving women with epilepsy found that, compared to women without epilepsy, there was a slightly higher risk of: spontaneous miscarriage, antenatal hemorrhage, hypertension-related disorders, fetal stunting, premature birth, need for induction of labor, cesarean section, and postpartum hemorrhage [5]. No differences were found between the two groups regarding perinatal death [5].

Due to an increased risk of fetal malformations in pregnant women with epilepsy, ultrasonography should be recommended and performed during pregnancy. Correct examination in the first and second trimester of pregnancy allows for the detection of fetal anatomical abnormalities. If a fetal defect is identified, therapeutic options during pregnancy and after birth should be evaluated. Parents should be given a choice of how to proceed [5,57].

Children of mothers with epilepsy taking AEDs demonstrate an increased risk of having a smaller weight in relation to gestational age, and therefore special attention should be given to fetal biometry in the third-trimester ultrasonography examination. In pregnant women treated with enzyme-inducing drugs (e.g., CBZ, PB) who are at risk of premature birth, the effectiveness of prevention of respiratory distress in infants may be limited due to an increased corticosteroid metabolism [5]. However, there is no evidence to suggest a clear benefit of corticosteroids at a larger dose and therefore routine doubling of a dose in cases of premature birth is not recommended [5].

## 14. Prophylaxis with Vitamin K

AEDs that are inducers of hepatic enzymes, such as CBZ, PHT, PB, primidone (PRM), OXC, TPM, are competitive inhibitors of prothrombin precursors, posing a risk of hemorrhage into body cavities and brain in neonates. Such complications have a high mortality rate of up to 30% [25]. To reduce this risk, it is recommended that pregnant women using AEDs, that induce hepatic enzymes, be given vitamin K at a dose of 20 mg per day during the last two weeks before delivery, and that 1 mg of vitamin K be given to the newborns [12,25]. Panchaud et al. [58] compared the influence of hepatic enzyme inducers on the risk of perinatal bleeding to that of other AEDs (VPA, LTG, LEV, PGB, GBP, clonazepam). The study was conducted on 11,572 pregnant women with epilepsy. A considerable fraction of patients on enzyme inducers were supplemented with vitamin K before delivery. Postpartum hemorrhage was evident in 2.6% (135/5109) in the group on enzyme inducers and in 3.6% (231/6463) in the group on other AEDs. The prevalence of neonatal bleeding did not differ significantly among the two groups. Also, data exist showing that prenatal vitamin K supplementation did not cause any significant difference in postpartum hemorrhage in women taking enzyme-inducing AEDs [59].

## 15. Delivery in Women with Epilepsy

Perinatal care for pregnant women with epilepsy should be provided in specialized centers that offer the highest level of perinatal and neurological services. A diagnosis of epilepsy is not an indication for planned cesarean section or induction of labor. There is no indication for earlier delivery in women with epilepsy without obstetric risk factors whose seizures are well controlled. Cesarean section may be considered for a small percentage of women with a significant increase in seizures, cluster seizures, and a high risk of status epilepticus. Obstetrical care in women with epilepsy does not differ from the commonly accepted principles of modern obstetrics [5].

Pregnant women with epilepsy should be informed that the risk of having a seizure during labor and after delivery is low, approximately 1–2%. Adequate pain management and appropriate care during labor should be provided to minimize risk factors for seizures, such as insomnia, stress, and dehydration. Administration of AEDs should be continued during labor as per the neurologist’s recommendations. If they cannot be administered orally, parenteral administration should be an alternative (VPA, LEV, PHT may be administered intravenously). Seizures during labor can lead to mother’s hypoxia (due to apnea during seizure), as well as fetal hypoxia and acidosis secondary to increased tension of the uterus. Adequate hydration and pain relief through epidural anesthesia reduce the risk of a seizure during labor. Nitrous oxide can also be used as an analgesic. Administration of pethidine is contraindicated because of the risk of a lowered seizure threshold. Early epidural anesthesia can minimize risk factors for seizures during labor, such as hyperventilation, sleep deprivation, pain, and stress. If general anesthesia is required, anesthetics such as pethidine and ketamine must be avoided (they lower the seizure threshold) [5].

There are no known contraindications to the use of any labor-inducers in women with epilepsy taking AEDs. There is no evidence that AEDs affect labor-inducing medications. It is important to note that risk factors such as stress, insomnia, and dehydration are exacerbated in women with prolonged induction and should be minimized [5].

Epilepsy and pregnancy do not constitute an indication for surgical delivery. The method of delivery is determined by the obstetric conditions and indications as well as the neurological condition of the patient [13,25].

## 16. Postpartum in Women with Epilepsy

Due to increased stress, lack of sleep, anxiety, and missing an AED dose, the immediate period after birth involves a high risk of more frequent seizures. Mothers should be supported in the postpartum period to minimize risk factors for seizures; in particular, they should be ensured a continuous 4–6 h period of sleep [5,60].

Many women receive higher doses of AEDs at the end of pregnancy compared to the doses before pregnancy. During the postpartum period, the physiological changes that occurred during pregnancy, such as increased renal and hepatic clearance and hemodilution, subside, resulting in a risk of high-dose AEDs toxicity to the newborn. If a drug dose had been increased during pregnancy, then the drug concentration should be determined within 10 days postpartum and the dose reduced accordingly to avoid postpartum toxicity. Urgent neurological assessment is required if symptoms of AED toxicity occur during puerperium (e.g., drowsiness, double vision, or balance disorders) [5,9,60].

The newborns delivered by women taking AEDs should be monitored for adverse effects (lethargy, feeding difficulties, excessive sedation or withdrawal symptoms—excessive excitability and crying) associated with exposure to AEDs in utero. Women with epilepsy and their children should be cared for by perinatal centers which have the capacity to determine AED concentrations and cooperate with neurological centers specializing in the care of patients with epilepsy [5,9,60].

Women with epilepsy display an increased risk of depression in the postnatal period compared to mothers without epilepsy. Symptoms of postnatal depression include lowered mood, fatigue, lack of appetite, feelings of tension, or anxiety. Early intervention can improve women’s quality of life [5,9,60].

## 17. Mortality of Women with Epilepsy in Pregnancy

The estimated mortality rate among women with epilepsy for the entire pregnancy and 42 days after delivery is approximately 10 times higher than in the general population. The risk of an epileptic woman dying during pregnancy is 2–3 times higher than during another period in her life [61,62].

The causes of increased mortality in women with epilepsy during pregnancy may be: discontinuation of medication, poor co-operation with the patient, non-compliance with medical instructions which may lead to an increased incidence of epileptic seizures, status epilepticus, and SUDEP syndrome [13].

## 18. Status Epilepticus during Pregnancy

The occurrence of status epilepticus during pregnancy of patients with epilepsy is a rare but life-threatening condition both for the patient and her child. In the EURAP study evaluating 3784 pregnancies, status epilepticus occurred in 21 (0.6%) patients, of whom 10 experienced convulsive status epilepticus. Although no instance of a woman dying was recorded, there was one case of a child dying due to the mother’s convulsive status epilepticus and three children born with severe defects [13].

## 19. Sudden Unexpected Death in Epilepsy (SUDEP) during Pregnancy

In the United Kingdom, 11 women with epilepsy died due to SUDEP during pregnancy or shortly after out of 13,978 maternities. They were mainly on LTG, which was typical for the United Kingdom prescribing practice [63]. Between 2010 and 2019, maternal death associated with SUDEP was evaluated in Japan. Six women died in this period, four being on monotherapy and two receiving no therapy with AEDs. The corresponding mortality rate was thus 0.066/100,000 individuals [64].

## 20. Breastfeeding by Women with Epilepsy

Women with epilepsy taking AEDs during pregnancy should be encouraged to breastfeed. Mothers should be informed that—according to current knowledge—the risk of cognitive complications is not increased in children exposed to AEDs contained in the milk of women who take such drugs [5,9,60]. While it is important to remember that all AEDs pass into the mammary gland and are excreted in milk, their concentrations in milk are much lower than in the blood serum of the lactating patient. Prohibiting breastfeeding may expose the newborn to the risk of developing withdrawal syndrome, which could be more dangerous than continued breastfeeding. If mothers receive high doses of PB, PRM, and benzodiazepines, the child may exhibit signs of drowsiness [5,9,60]. In such instances, inhibition of lactation should be considered. A prospective study revealed that the psychomotor development of children who were exposed to AEDs in utero and breastfed after birth was better at 6 and 18 months compared with children who were not breastfed or who were breastfed shorter than 6 months [5,9,60]. Women who experienced no epileptic seizures during pregnancy can feed their child without any assistance, whereas those who have had seizures should breastfeed in the presence of a family member or medical personnel, if possible [5,65]. AEDs that are considered safe to a child whilst breastfeeding are: PHT, VPA, CBZ; moderately safe: LTG, OXC, LEV, TPM, GBP, PGB, vigabatrin, tiagabine (TG); likely to be dangerous: PB, PRM, benzodiazepines (BD), ethosuximide, ZSM, felbamate [39,41]. No precise data are available for other AEDs—perampanel, LCM, eslicarbazepine [65].

The NEAD study compared the IQ evaluated in six-year-old children of mothers receiving AEDs. Breastfed children demonstrated higher IQ levels compared to non-breastfed children (108 vs. 104) [39]. In the group of breastfeeding mothers taking CBZ (107 vs. 105), VPA (106 vs. 94), or LTG (113 vs. 110), children’s IQ level was higher than in the group of women taking the very same drugs but not breastfeeding and it was lower in the children of mothers taking PHT (104 vs. 108) [65].

The concentrations of most AEDs in breast milk are significantly lower than in blood serum. For instance, the concentration of VPA does not exceed 10% of that encountered in serum. Then, PHT, CBZ, PHT and LTG are in the range of 20, 40, 50 and 61%, respectively. On the other hand, newer AEDs, OXC, TPM, and LEV reach 80, 86, and 100% of their serum concentrations [1]. Regardless of the above data, women with epilepsy are in general less likely to choose to breastfeed at all and are more likely to breastfeed for a shorter period compared to other mothers [12]. Results reported by Birnbaum et al. [66] strongly recommend breastfeeding to mothers with epilepsy. Interestingly, 49% of all AED (CBZ, LEV, LTG, OXC, TPM, VPA, ZSM) concentrations in infant blood samples did not exceed the lower limit of quantification. When the median percentage of infant-to-mother AED concentration was considered, it ranged from 0.3% (range of 0.2–0.9%) to 44.2% (35.2–125.3).

## 21. Information for Breastfeeding Mothers Who Receive AEDs

- Breastfeeding is safe and should be recommended for the period of at least 6 months, preferably 12 months.
- Drugs considered safe include: PHT, VPA, CBZ.
- Drugs considered moderately safe: LTG, OXC, LEV, TPM, GBP, PGB, vigabatrin, TGB.
- Caution recommended: PB, PRM, benzodiazepines, ethosuximide, ZSM, felbamate.
- No information available on some newer AEDs: perampanel, LCM, eslicarbazepine.

Information on the safety of drugs for breastfeeding women can be found at: http://toxnet.nlm.nih.gov/newtoxnet/lactmed.htm (last accessed on 12 December 2021). The information is updated monthly [65].

## 22. Focus on VPA

### 22.1. Generic Adverse Effects

Chronic administration of VPA in patients with epilepsy may be associated with reversible or irreversible hepatotoxicity [67]. In about 5–10% of patients, mild elevation of serum aminotransferases and transaminases is encountered, however, with no progression to serious liver injury. Mostly, children within the first 3 months of therapy with VPA may suffer from irreversible liver toxicity at the rate of 1/40,000 patients. Also, within the initial 6 months of VPA therapy, hyper-ammonemia may occur in 16–80% of patients. Hyperammonemia is mostly asymptomatic and rarely may result in encephalopathy (lethargy, disorientation, drowsiness). Probably, the inhibition of mitochondrial β-oxidation is associated with fatty liver degeneration, but the incidence of this adverse effect is quite low (1/37,000). Apart from hepatotoxicity, VPA may induce tremor, nausea, and dizziness. In the pediatric population, this AED was reported to produce weight gain (in 16% of patients), nausea (9%), and increased appetite (8%) [67].

### 22.2. Neurodevelopmental Risks

The available evidence underlines that VPA is the most dangerous AED in terms of teratogenicity and the risk of malformations is positively correlated with the dosage of this AED. One of the main mechanisms of VPA action is the potentiation of GABA-mediated events [68]. This AED has been also documented to inhibit histone deacetylases so through epigenetic modifications, it may influence the expression of a number of genes involved in cell cycle control and cellular differentiation [69]. Evidently, the risk of malformations is high starting from the daily dose of 1.5 g [70]. However, maternal VPA exposure can also lead to ASD [54]. This evident hazard has been extensively studied in animals and eventually, a rat model of VPA-induced ASD was elaborated [71]. VPA-intrauterine exposed male rats were tested for the occurrence of behavioral specific alterations which were: (i) lower pain susceptibility and elevated sensitivity to non-painful stimuli, (ii) reduced acoustic prepulse inhibition, (iii) decreased exploration associated with locomotor hyperactivity and stereotypic-like behaviors, and (iv) significant reduction in social interactions and prolonged latency to social behaviors. All these aberrant behaviors were evident in the pre-pubertal period [71]. Interestingly, in male rats exposed prenatally to VPA (a single intraperitoneal injection of VPA at 600 mg/kg to females on gestational day 12.5), there was a reduced expression of proenkephalin mRNA in the dorsal striatum and nucleus accumbens and these animals exhibited increased anxiety and diminished conditioned place aversion to naloxone. However, learning and memory were not affected in VPA rats [72]. Considering the male VPA offspring, they also differed in terms of endocrinological and immunological data. Basal concentration of corticosterone was elevated, the thymus weight and proliferative response of splenocytes to concavaline A were reduced. Besides this, the ratio of interferon gamma/interleukin 10 was lowered and peritoneal macrophages produced more nitric oxide [73]. On the other hand, female VPA rats exhibited fewer aberrant behavioral and immunological effects—increased stereotypic-like behaviors and reduced interferon gamma/interleukin 10 ratio being only observed [73].

Prenatal exposure to VPA (600 mg/kg on gestational day 12.5) also resulted in profound reductions in mRNA expressions of genes related to social behavior in young male and female adult rats [74]. These are genes for 5-hydroxytrypatmine receptors, brain-derived neurotrophic factor (BDNF), and neuroligin3. The expression of hippocampal glutamic acid decarboxylase (an enzyme synthesizing GABA) was also reduced. On the other hand, in both male and female offspring, significant increases in hippocampal proinflammatory cytokines (interleukin-1β, tumor necrosis factor-α) were noted. Alterations in gene expressions, concentrations of proinflammatory cytokines, and activity of glutamic acid decarboxylase were evident in rats that manifested, as described previously [72], characteristic behavioral symptoms.

Kinjo et al. [75] used a different experimental approach in that they injected pregnant rats with daily intraperitoneal VPA (100 or 200 mg/kg) starting from gestational day 12.5 until birth. On postnatal day 29, male offspring were administered bromodeoxyuridine (to study cell proliferation in the dentate gyrus), and at postnatal day 30, the animals were tested in a battery of tests, including open field, elevated plus-maze, and Y-maze tests. Brain malformations were observed in 66.6% of VPA 200 mg/kg rats, which also demonstrated locomotor hyperactivity recorded in the open field test. Also, irrespective of the VPA dose, the rats stayed longer in the open arms when studied in the elevated plus-maze test. In the dentate gyrus, the number of bromodeoxyuridine-positive cells was significantly increased in a dose-dependent manner. Another study on this issue differed only in that the experimental studies were performed on postnatal day 150 and bromodeoxyuridine was given a day before [76]. Interestingly, no aberrant behaviors were noted whilst the VPA animals still differed in the number of bromodeoxyuridine-positive cells in the dentate gyrus. However, in contrast to the postnatal day 30, the number of cells was decreased by 50% [76]. VPA (200 mg/kg, i.p.) was also given at gestational day 14 (when the genesis of cortical upper layer neurons takes place) to pregnant mice [77]. Male offspring, evaluated between postnatal 8 and 9 weeks, showed typical behavioral abnormalities (impaired social interaction, increased motor activity, and learning deficits) and disturbed neural activity associated with enhanced neuronal density in the prefrontal cortex, but not somatosensory area [77]. Kawada et al. [78] administered pregnant mice with VPA (500 mg/kg) at 9.5 days gestation and as expected, the male offspring (10-week-old) presented characteristic behavioral disturbances (less exploratory activity, increased anxiety-like behavior, enhanced aggression). The authors decided to study endoplasmic reticulum stress and expression of genes involved in the control of neuronal differentiation at postnatal day 1 in the cerebral cortex and hippocampus. It turned out that the endoplasmic reticulum stress marker, glucose-regulated protein 94, was more expressed in these brain regions of VPA mice. Moreover, *Hes1* (inhibiting neuronal differentiation and maintaining neural stem cells in the undifferentiated state) and *Pax6* (enhancing the proliferation of neural stem cells and promoting maintenance of neural stem cells in the undifferentiated state) genes were less expressed whilst the expression of *Math1* and *Neurogenin* mRNA (genes raising the differentiation of neuronal lineage) was increased in the cerebral cortex of VPA mice. No such alterations were found in the hippocampus [78].

The exposure to maternal VPA (200 or 300 mg/kg on gestational days 26 and 29) has been also tested in non-human primates providing valuable data on the neurodevelopmental and behavioral correlates [79]. In the offspring brains, significant reductions in NeuN-positive neurons (in the prefrontal cortex and cerebellum) and Ki67-positive proliferating neuronal precursors (in cerebellar external granular layer) were found. However, GFAP-positive astrocytes were increased in the prefrontal cortex [79]. Aberrant behavior in the ASD model in VPA rats (600 mg/kg on gestational day 12.5) was also correlated with the increased concentration of blood microRNA (miR138-5p) in 30-day-old VPA offspring [80].

Considering that most studies of ASD in experimental animals were brought about in males, Dos Santos et al. [81] have decided to compare the effects of prenatal VPA exposure (600 mg/kg on gestational day 12.5) on behavioral abnormalities in male and female offspring (housed either in standard or enriched cages) with the use of hippocampal-related tasks in five-month-old animals. Also, the number and morphometry of microglial cells located in the molecular layer of the dentate gyrus were evaluated. VPA mice exhibited abnormal exploratory behaviors associated with the reduced risk assessment, females showing more aberrant behavior. Both, the volume of the molecular layer and the number of microglial cells were considerably higher in VPA mice from standard cages. Mice housed in enriched cages presented less intense behavioral and cellular abnormalities [81].

Quite different results were obtained when pregnant rats received a moderate dose of VPA (350 mg/kg on gestational day 13). VPA rats were tested on postnatal days 28 (early adolescence), 42 (late adolescence), and 75 (young adulthood). It is of interest that VPA rats exhibited more social investigation and play fighting compared to controls. These particular behaviors tended to increase in late adolescence [82]. Evidently, these data are in contrast to the previous data from VPA rats exposed to 600 mg/kg of this AED [71]. Cohen et al. [82] also provided evidence that transcriptomic alterations were recorded in VPA rats in three brain regions—anterior amygdala, cerebellar vermis, and orbitofrontal cortex. Consequences of intrauterine exposure to VPA in rodent offspring are shown in Table 2.

Considering that maternal exposition to VPA may be responsible for neurodevelopmental disorders, an intriguing question arises whether paternal VPA exposure may induce comparable alterations in offspring [83]. Male mice (8 weeks old) were given VPA at daily doses of 30 or 100 mg/kg) for 4 weeks. For the last week of VPA administration, prior to natural mating, each male was put into a cage with two naïve female mice. The offspring were tested on postnatal day 56. Behavioral data indicate that paternal VPA exposure led to an impairment of object cognitive memory and reduction of N-methyl-D-aspartate agonist-induced locomotor hyperactivity. However, sensorimotor gating was only disturbed in female offspring. Brain histone H3 concentration was down-regulated [83].

Interestingly, ASD in offspring resulting from prenatal exposure to VPA in pregnant rats can be effectively prevented by maternal folic acid (4 mg/kg) supplementation. Possibly, this positive effect of folic acid may be dependent upon restoring the balance between synaptic proteins associated with excitatory and inhibitory neurons [84].

An important question arises whether prenatal exposition to VPA may be associated with ASD in children born to mothers with epilepsy in view of the above experimental data. Wiggs et al. [54] have analyzed 14,614 children who were born between 1996 and 2011. During the first trimester, 22.7% of mothers were taking one AED and 9.7% were on CBZ, 6.8%—on LTG and 4.8% on VPA. Only diagnoses made in children above 2 years of age were considered. The results indicate that there was an increased risk for the development of ASD in children exposed prenatally to VPA and the risk was not confirmed after exposition to either LTG or CBZ. Veroniki et al. [25], although confirming the data on VPA, also provide evidence that prenatal LTG and OXC may also produce an increased occurrence of ASD. Noteworthily, a similar risk associated with prenatal VPA was also reported for ADHD [54]. Out of 580 VPA children, 4.8% developed ADHD and they had a 48% elevated risk of ADHD in comparison to children unexposed to VPA [85]. However, the risk of ADHD in children exposed to other AEDs (CBZ, clonazepam, LTG, OXC) was not statistically greater when compared with children not exposed prenatally to AEDs [85].

Recently, Honybun et al. [86] have addressed the question of whether prenatal VPA exposure and related neurodevelopmental abnormalities are sex-dependent. The study comprised 121 children of whom 54 (28 males, 26 females) were prenatally exposed to VPA and 67 to other AEDs. The mean dose of VPA was 644 ± 310 mg daily. On the whole, significantly higher ASD scores were shown in males as regards all AEDs. The sex-dependence, however, was not found in children exposed prenatally to VPA. Moreover, no dose–response relationship was evident between prenatal exposure to VPA and ASD symptoms. Again, males presented higher ADHD scores in comparison to females when exposure to all AEDs was considered and this relationship was no more observed in relation to VPA [86].

### 22.3. Mechanism Leading to Fetal Malformations

As already mentioned, in utero exposition to VPA may lead to congenital malformations—cardiac, craniofacial, limb, neural tube, orofacial, and skeletal malformations [87]. Existing animal data have provided a number of mechanisms probably responsible for the teratogenicity of VPA.

One possible mechanism for teratogenicity may result from the direct mechanism of action of VPA. This AED was injected on embryonal day 8 at 400 mg/kg. To evaluate the histone acetylation in the embryo homogenates, embryos were extracted 1 h after drug administration. VPA-induced histone hyperacetylation was particularly evident in the caudal neural tube and somites as visualized by immunohistochemistry [88]. Malformations in fetuses at term were observed in the axial skeleton [88]. Further, in another experiment [89] VPA was given in the dose of 400 mg/kg on gestational day 9 and the extraction of embryos followed 1, 3, 6, and 24 h after the drug administration. Similar to the previous experiment, histone acetylation was enhanced and the maximum effect was seen 3 h after VPA injection. Also, histone methylation (at histone H3 lysine 4) was increased whilst at histone H3 lysine 9—reduced. Results from immunohistochemistry indicate that enhanced histone acetylation was present in the neuroepitelium, heart, and somites. Reduced histone methylation (H3 lysine 9) was evident in the neuroepitelium and somites and increased (H3 lysine 4)—in the neuroepitelium [89]. The same authors, under almost the same experimental conditions (an additional time period of 0.5 h was added for the extraction of embryos), evaluated the effects of prenatal VPA on DNA damage and downstream alterations in cell cycle inhibitors and apoptosis [90]. Western blotting and immunohistochemistry were used to evaluate the expression of gammaH2AX (a biomarker for DNA double-strand breaks), p27(KIP1) (a multifunctional cyclin-dependent kinase inhibitor), and cleaved caspase-3. In VPA embryo homogenates, after 0.5 h gammaH2AX expression was significantly elevated and then decreased. Expression of p27(KIP1) and cleaved caspase-3 was increased 3 and 6 h after VPA administration. All these alterations were found in the embryo head [90].

Transgenic pKZ1 mice are used in order to examine somatic intrachromosomal recombination in the presence of agents damaging DNA [91]. Lamparter and Winn [92] decided to study the effect of VPA (500 mg/kg administered subcutaneously to pregnant pKZ1mice on gestational day 9) on the frequency of intrachromosomal recombination and the expression of genes responsible for DNA double-strand breaks repair, by nonhomologous end joining (NHEJ) or homologous recombination (HR), in embryos. The embryos were explanted 1, 3, 6, and 24 h after VPA administration. As a surrogate measure of recombination, *lacZ* transcript levels were used. Elevated intrachromosomal recombination (1.62-fold) was found in the embryo head after 6 h from VPA exposure. The mRNA expression of the NHEJ repair gene, *Xrcc4*, was significantly changed in no studied tissues or time points. The expression of the HR repair gene, *Rad51*, was significantly reduced in the embryo head (1.52-fold) 1 h after VPA administration. In contrast, *Rad51* expression was significantly increased in the head (2.0-fold), heart (1.6-fold), and trunk (1.92-fold) after 6 h. By 24 h, the expression of both repair genes returned to control values. After 1 h, expression of other HR genes, *Brca1* and *Brca2*, was decreased in the head (1.96- and 1.72-fold, respectively) and trunk (1.45- and 1.69-fold). Following 3 h after VPA exposure, expression of both genes was significantly elevated in the heart and trunk tissue. Considering the head tissue after 6 h, *Brca1* expression did not differ from the control values whilst that of *Brca2* was significantly increased (1.47-fold). Again, after 24 h there were no significant changes in the expression of these HR genes. Finally, after 3 h, cleaved caspase-3 and PARP protein expressions were significantly elevated in the embryo head (10.13- and 18.06-fold, respectively), heart (2.4- and 3.24-fold), and trunk (5.59- and 11.45-fold) [91].

Lin et al. [93] have compared teratogenic effects of VPA and its amide derivative—valnoctamide in mouse embryos. Pregnant mice were given both AEDs (at 1.8 or 2.7 mmol/kg) on gestational day 8, and the evaluation of teratogenicity was carried out on embryonal day 18. The expression of genes involved in neurogenesis and neural stem cell differentiation was estimated 4 h after exposure to VPA (2.7 mmol/kg) or valnoctamide (2.7 mmol/kg). The following fetal defects were observed: visceral, missing skull bones, and fused vertebrae. Visceral defects were dose-dependent whilst the remaining abnormalities were induced by the exposure to VPA at its higher dose. Valnoctamide was evidently less teratogenic. The expression of three genes, *Mtap2* (involved in early neural development [94]), *Bmp8b* (controlling morphogenesis of epithelial tissue [95]), and *Stat3* (responsible for cell growth and migration [96]) was significantly elevated in fetuses exposed to VPA. *Heyl* (involved in neurogenesis, somitogenesis, and organogenesis [97]) was in contrast downregulated in the fetal tissue exposed to VPA [93].

Experiments obtained from mouse post-implantation embryos exposed to VPA (0.6 mM which corresponds to 400 mg/kg in vivo) point to oxidative stress as a factor involved in neural tube defects [98]. Concentrations of reactive oxygen species were significantly increased in the head region. In parallel, apoptotic markers were also elevated [98]. In another in vitro study, mouse embryonic forelimbs (gestation day 12) were excised, cultured, and exposed to VPA for 3, 6, 12, and 24 h [99]. Then, their influence on *p53* (a gene involved in embryonic development [100]) signaling (target genes and proteins) and apoptosis markers during midorganogenesis was evaluated. In VPA limbs, p53 was hyperacetylated and the expression of its target genes, *Survivin/Birc5* and *Bcl2*, was decreased whilst that of *p21/Cdkn1a*—increased. Further, apoptosis and DNA damage markers were elevated [99].

As already mentioned, folic acid supplementation may prevent fetal malformations induced by VPA or other AEDs [49,50]. Experimental data provide further evidence clearly indicating that folic acid distinctly counteracted VPA-produced developmental neurotoxicity in zebrafish embryos [101]. Exposure of embryos to VPA at 1–30 µmol/L resulted in a reduction of the midbrain size and an increase in the midline gap of the hindbrain. Supplementation with folic acid at 3 or 30 µmol/L reduced VPA (3 µmol/L)-induced structural brain defects. Apart from brain defects, VPA exposure also led to a decrease in the number of neuronal progenitor cells and disturbed neurite spouting of the secondary motor neurons. In the presence of folic acid, the neurotoxic action of VPA was significantly diminished [101].

## 23. Conclusions

This review presents an overview of the issues that affect women with epilepsy during pregnancy, labor, and early childcare. Knowing these issues may enable proper therapeutic management and increase the chances of normal pregnancy and delivery in women with epilepsy, as well as the normal development of a child.

The following should be taken into consideration:

- Over 90% of women with epilepsy may deliver healthy children.
- Pregnancy planning is the safest solution for a woman and her future child.
- If changing AEDs is necessary, it should be done during the preconception period.
- The use of a single AED at the optimal dose for the patient is preferred, consistent with the type of seizures or epilepsy syndrome and at the lowest possible teratogenic risk.
- Women who had no seizures nine months before conception have a greater than 80% chance of not experiencing seizures during pregnancy.
- In most women, the frequency of seizures in pregnancy does not change significantly, but in some women, an increase in the dose of AEDs is recommended to ensure good seizure control, especially during the third trimester.
- Regular intake of AEDs, as recommended by a physician, is essential to maintain good seizure control.
- It is recommended to administer a prophylactic dose of 4–5 mg of folic acid per day 3 months before pregnancy and during the first trimester.
- Most women can have a natural childbirth. Indications for cesarean section should be limited to the occurrence of frequent tonic-clonic seizures or other types of seizures that may disrupt the course of labor.
- In most cases, the benefits of breastfeeding are considered to outweigh the possible risks associated with the adverse effects of AEDs on the child.

As already mentioned, some pregnant women with epilepsy are prescribed AED combinations if monotherapy is not sufficient. VPA has been associated with a greater risk of malformations in combinations with other AEDs [102]. For example, in the case of LTG + VPA, the malformation risk is 9.1% whilst for LTG combined with an alternative AED—the risk of 2.9% was estimated. A comparable dependence was noted in the case of CBZ, the risk of 15.4% being observed with combinations of this AED with VPA. When VPA was substituted by any other AED, the risk went down to 2.5% [102]. According to Vajda et al. [103], pregnant women with epilepsy may be prescribed AED combinations with a low malformation risk provided that VPA and TPM are avoided. This indicates that the risk of malformations seems dependent on the highest risk AEDs rather than on the fact of combining AEDs. Considering hyper-additive anticonvulsant combinations of AEDs (data from animal and clinical studies), many of them include VPA or TPM. For instance, LTG + VPA, CBZ +VPA, CBZ + TPM, LEV + VPA [104]. These AED combinations must be, however, avoided in pregnant women. Anyway, there are still relevant AED combinations available without VPA or TPM—for example, CBZ + GBP, or OXC + LEV [104] and these may be recommended for pregnant women with epilepsy when monotherapy fails. A combination of LEV + LTG, although hyper-additive in terms of seizure protection, cannot be recommended due to the high risk of major malformations as shown in Table 3. Certainly, antagonistic AED combinations need to be avoided as ones providing irrelevant seizure control. Solid experimental data and some clinical studies point to CBZ + LTG as an antagonistic AED combination [104] which, although relatively safe for the fetus, may not provide significant seizure control. Taking into consideration the results of an in vitro study carried out on human embryonic stem cells, GBP seems much safer compared to CBZ, LEV, LTG, or TPM. These AEDs produced considerably more DNA damage than GBP [105]. Thus, when polytherapy cannot be avoided in pregnancy then combined treatments with the use of GBP may be safer. It should be particularly accentuated that many AED combinations with GBP have been shown to exert synergistic anticonvulsant effects with no neurotoxicity being observed [106].

VPA teratogenicity has been well documented and one of the major mechanisms involved in this negative effect seems inhibition of histone deacetylases [88]. Also, VPA may be teratogenic as a folate antagonist [107]. Valnoctamide (an analog of VPA) applied in an equimolar dose was much less teratogenic than VPA, which may suggest that the analog is not an inhibitor of histone deacetylases or a folate antagonist. In fact, other analogs of VPA have not been shown to share these mechanisms of action [107,108].

Although folate usage during pregnancy is recognized and recommended, the malformation rate over two decades (1997–2017) has not significantly changed (according to the Kerala Registry of Epilepsy and Pregnancy [109]. In the same time period, the use of folate was increased in the pre-pregnancy month and first trimester. Noteworthy, newer AEDs (LTG, LEV, OXC, TPM) were actually preferred, older generation AEDs (PB, PHT, clonazepam) being less frequently applied in the last seven years. However, the use of CBZ or VPA was not reduced in pregnant women. In the authors’ opinion, this unexpected result may be due to VPA and/or increased use of TPM [109].

Intrauterine exposure of rodents to VPA may serve as a model of ASD. Usually, VPA is given on gestational day 12.5 at a high dose of 600 mg/kg. The rodent offspring present a number of aberrant behaviors, including disturbed social interactions. The results obtained by Cohen et al. [82] need to be especially accentuated. They injected pregnant rats with a much lower dose of VPA (350 mg/kg) and the offspring presented behavioral alterations totally different from those characteristic for VPA at 600 mg/kg. According to the authors, they can be hardly considered homologous to ASD in humans. To the degree, the experimental data can be transferred to clinical conditions, the results of this study may indicate that low doses of VPA during pregnancy actually reduced the risk of ASD and malformations. There is a consensus that the use of VPA during pregnancy or even in women of childbearing age has to be avoided [87]. Although animal data strongly indicate that paternal exposure to VPA results in obvious behavioral abnormalities [83], a population-based cohort study does not confirm this result. Only a tendency for increased rates of autism or intellectual disability was noted [110].

## Figures and Tables

**Table 1:** Dose-dependent teratogenic effect of the most commonly used AEDs.

Dose of an AED	EURAP Registry [10]		British Registry [33]
	Up to 2 Months	Up to 1 Year	Up to 6 Weeks
VPA			
600 mg/day	ND	ND	5.0% (N = 476)
<700	4.2% (N = 431)	5.6%	ND
>1000	ND	ND	10.4% (N = 297)
≥700–1500	9.0% (N = 480)	10.4%	ND
≥1500	23.2% (N = 99)	24.2%	ND
			
CBZ			
<400 mg/day	1.3% (N = 148)	3.4%	ND
0–≤500	ND	ND	1.9% (N = 729)
≥400–<1000	3.2% (N = 1047)	5.3%	ND
>500–≤1000	ND	ND	2.7% (N = 739)
≥1000	7.7% (N = 207)	8.7%	5.3% (N = 170)
			
LTG			
0–≤200 mg/day	ND	ND	2.1% (N = 1143)
>200–≤400	ND	ND	2.4% (N = 665)
<300	1.7% (N = 836)	2.0%	ND
≥300	3.6% (N = 444)	4.5%	ND

Infants were exposed to AEDs during the first trimester. AEDs: antiepileptic drugs, CBZ: carbamazepine, LTG: lamotrigine, VPA: valproate, N: number of infants, ND: not determined.

**Table 2:** Consequences of intrauterine exposure to valproate (VPA) in rodent offspring.

VPA Intrauterine Exposure in Rodents	Effects in Male Offspring Unless Stated Otherwise	Reference
Single i.p. injection at 600 mg/kg on gestational day 12.5 in rats	Pain susceptibility ↓ Acoustic prepulse inhibition ↓ Exploration ↓ with locomotor hyperactivity Social interactions ↓	[71]
The same as above	Expression of proekephalins mRNA ↓ in dorsal striatum and nucleus accumbens Anxiety ↑ Conditioned place aversion for naloxone ↓	[72]
The same as above	Basal corticosterone concentration ↑ Thymus weight ↓ Proliferative response of splenocytes to convacaline A ↓ Interferon gamma/interleukin 10 ratio Production of nitric oxide by peritoneal macrophages ↑	[73]
The same as above	mRNA expressions of genes related to social behavior ↓ (both in males and females) Hippocampal glutamic acid decarboxylase ↓ Hippocampal proinflammatory cytokines ↑ (also in females)	[74]
VPA at 200 mg/kg i.p. daily, starting on gestational day 12.5 until birth in rats	Brain malformations in 66.6% at postnatal day 30 Locomotor activity in the open field test ↑ Time spent in open arms in the elevated plus-maze test ↑ Number of bromodeoxyuridine-positive cells ↑ in the dentate gyrus	[75]
The same as above	No aberrant behavior at postnatal day 150 Number of bromodeoxyuridine-positive cells ↓ in the dentate gyrus	[76]
VPA (200 mg/kg, i.p.) administered on gestational day 14 to pregnant mice	Impaired social interaction, locomotor activity ↑, learning deficits Impaired neuronal activity with neural density ↑ in the prefrontal cortex	[77]
VPA (500 mg/kg) given on gestational day 9.5 to pregnant mice	Less exploratory activity, increased anxiety-like behavior, enhanced aggression Expression of glucose-regulated protein 94 ↑ (endoplasmic reticulum stress marker) in the cerebral cortex and hippocampus Expression of *Hes1* gene ↓ (involved in inhibiting neuronal differentiation) Expression of *Pax6* gene ↓ (associated with enhanced proliferation of neural stem cells) Expression of *Math1* and *Neurogenin* genes (increasing the differentiation of neuronal lineage) ↑ in the cerebral cortex	[78]
VPA (600 mg/kg) on gestational day 12.5 to pregnant rats	Abnormal exploratory activity, even more expressed in female offspring Volume of the molecular layer and number of microglial cells ↑ in the dentate gyrus	[81]
VPA (350 mg/kg) on gestational day 13 to pregnant rats	Social investigation and play fighting ↑ Transcriptomic alterations in anterior amygdala, cerebellar vermix, and orbitofrontal cortex	[82]
VPA (600 mg/kg) on gestational day 12.5 to pregnant rats	Aberrant behavior Blood concentration of miR138-p ↑	[80]
VPA (300 mg/kg) on gestational days 26 and 29 to pregnant cynomolgus monkeys	NeuN-positive neurons ↓ in the prefrontal cortex and cerebellum Ki67-positive proliferating neuronal precursors ↓ in cerebellar external granular layer GFAP-positive astrocytes ↑ in the prefrontal cortex	[79]

↑: increase, ↓: decrease.

**Table 3:** Percentage of major malformations with different drug combinations as reported by two epilepsy and pregnancy registries.

AED Combination	North American AED Pregnancy Registry [102]	British Registry [34] (N = 367)
LTG + VPA	9.1% (N = 55)	ND
LTG + other AEDs	2.9% (N = 450)	ND
		
CBZ + VPA	15.4% (N = 39)	ND
CBZ + other AEDs	2.5% (N = 326)	ND
		
LEV + LTG	ND	1.8%
LEV + VPA	ND	6.9%
LEV + CBZ	ND	9.4%

LEV: levetiracetam. For other abbreviations see the legend of Table 1.

## Data Availability

Not applicable.
